# The Effect of the Species Source of Muscle and/or Digestive Enzymes on the Utilization of Fish Protein Hydrolysates as a Dietary Protein Source in First Feed for Larval Walleye (*Sander vitreus*)

**DOI:** 10.3390/ani14172493

**Published:** 2024-08-28

**Authors:** Giovanni S. Molinari, Michal Wojno, Genciana Terova, Macdonald Wick, Hayden Riley, Jeffery T. Caminiti, Karolina Kwasek

**Affiliations:** 1 Center for Fisheries, Aquaculture, and Aquatic Sciences, Southern Illinois University, Carbondale, IL 62901, USA; giovanni.molinari@siu.edu (G.S.M.);; 2 Department of Biotechnology and Life Sciences, University of Insubria, 3, 21100 Varese, Italy; 3 Department of Animal Science, The Ohio State University, Columbus, OH 44691, USA; 4 Department of Biological Sciences, University of New Hampshire, Durham, NH 03824, USA

**Keywords:** Walleye, larval nutrition, protein hydrolysate, species-specific protein

## Abstract

**Simple Summary:**

Due to the underdeveloped digestive tracts of larval fish, predigested fish protein has been included in feeds to help increase dietary protein utilization. The predigested protein has been produced from various protein sources and broken down using different methods. This has ultimately led to variable results when included in larval diets, and there is still a lack of an optimal dry diet for larval fish. This study tested the use of adult Walleye muscle as a protein source to provide the optimal dietary amino acid composition for same-species larvae. The muscle was also broken down with enzymes from the adult Walleye gut to predigest protein in a way that mimics the digestion of live Walleye. This protein source was tested against protein produced from muscle and/or enzymes from Nile Tilapia, as a non-species-specific source. The dietary inclusion of predigested protein produced from same-species muscle and digestive enzymes led to increased growth and amino acid absorption in larval Walleye. However, reduced survival in fish fed with the predigested protein suggests that further research is required to improve the pellet stability of the larval diets and reduce the loss of nutrients to the water.

**Abstract:**

Fish protein hydrolysates used in larval diets have been prepared from a variety of fish species, with different enzymes used to hydrolyze the protein. This study’s objectives were to determine the effect of the dietary inclusion of fish muscle hydrolysates obtained from species-specific muscle/enzymes—versus hydrolysates produced from muscle/enzymes of a different species—on the growth performance, survival, skeletal development, intestinal peptide uptake, and muscle-free amino acid (FAA) composition of larval Walleye (*Sander vitreus*). Eight protein products were obtained for this study, comprising an unhydrolyzed and hydrolyzed product from each combination of muscle/enzymes from Walleye and Nile tilapia (*Oreochromis niloticus*). Four diets were produced, and the dietary protein was provided in a 50/50 ratio of unhydrolyzed and hydrolyzed protein from the respective muscle/enzyme combination. Four groups were fed one of the corresponding formulated diets, and two groups of larvae, fed a commercial starter diet and *Artemia*, respectively, served as reference groups. Larval Walleye fed the diet containing protein produced with the species-specific muscle and enzymes had a significantly higher weight after the study—30% higher than any other group. A significant interaction effect between muscle and enzyme sources on the growth of Walleye larvae was observed. The species-specific combination also led to a significant increase in postprandial FAA and indispensable amino acid concentrations in muscle. No significant differences were observed between the hydrolysate-fed groups in survival, deformity occurrence, or peptide uptake. Each hydrolysate-based diet significantly reduced skeletal deformities and survival compared to the commercial diet. The results of this study suggest that species-specific muscles and enzymes produce a more optimal dietary protein source for larval fish than non-species-specific products. Further research should focus on improving the physical properties of the formulated diets to reduce possible leaching of hydrolyzed protein and improve the survival of fish larvae.

## 1. Introduction

To improve the utilization of dietary protein in fish larvae with underdeveloped digestive tracts, protein hydrolysates have been added to formulated diets as a substitute for intact protein. Hydrolysates from purified protein sources, such as casein [1,2], and those from more practical ingredients, such as fish [3,4,5] and plant meals [6], were investigated. Among the hydrolysates tested, fish protein hydrolysates appear to be the most suitable for fish larvae, particularly due to their complete amino acid profile and palatable properties [7,8]. Previous studies have compared the use of fish protein hydrolysates with hydrolysates from other animal sources, such as shellfish and invertebrates [6,9]. Although fish protein hydrolysates are the most efficient [6], research comparing the use of protein hydrolysates from different species is limited. Given the significant differences in proximate composition, amino acid profile, and overall quality of muscle protein between fish species [10,11], the species’ protein source appears to be critical in producing an ideal protein hydrolysate for fish larvae. 

Chemical hydrolysis and enzymatic hydrolysis are the two main methods used to produce hydrolysates, with enzymatic hydrolysis being preferred to obtain a higher nutritional value of the protein hydrolysate and the specific peptide-bond cleavages producing more consistent amino acid profiles [12,13,14]. Typically, protein hydrolysates are produced with commercial enzymes, such as Protease N^®^, Alcalase^®^, and Neutrase^®^, or with enzymes extracted from plants, such as papain, bromelain, and ficin [11,15,16]. The selection of these enzymes is important because previous studies have shown that hydrolysis of the same muscle with different enzymes has significant effects on its antioxidant properties, nutritional quality, degree of hydrolysis, and amino acid composition [11,17,18,19]. In addition to commercial enzymes, studies have investigated the use of endogenous digestive enzymes from fish viscera for the production of hydrolysates [16,20,21,22,23]. These digestive enzymes are considered more efficient for the production of protein hydrolysates and would reduce the costs associated with the use of commercial enzymes [15].

The species source of endogenous digestive enzymes is crucial for the production of an optimal hydrolysate, as the digestive physiology of the individual species differs considerably. Carnivores are characterized by larger stomachs, shorter intestines, and a higher content of proteases [24]. In contrast, the digestive tracts of omnivores/herbivores tend to have longer intestines, with smaller (or absent) stomachs, and higher levels of carbohydrases [25,26]. The use of species-specific digestive enzymes appears to be the most effective hydrolysis method to obtain a high-quality hydrolysate for fish larvae, as mimicking the in vivo digestion of proteins compensates for their lower proteolytic activity. This method of using species-specific enzymes for in vitro hydrolysis has proven to be successful [22]. 

In this experiment, we studied fish protein hydrolysates from the muscle of two different fish species, which were hydrolyzed with endogenous digestive enzymes from these species. We hypothesized that the combination of species-specific muscle and digestive enzymes would yield a hydrolysate that had the ideal amino acid composition in a form best suited for uptake and utilization by larval fish. The objective of this study was to determine the effects of muscle and endogenous digestive enzyme source on the production and dietary inclusion of fish protein hydrolysates and their effects on the following in larval Walleye (*Sander vitreus*): (1) growth performance and survival; (2) the occurrence of skeletal deformities; (3) the expression of the intestinal peptide transporter PepT1; and (4) the postprandial pool of free amino acids (FAA) in muscle, which serves as an indicator of dietary amino acid availability.

Walleye are considered cool-water carnivorous and stomach-possessing species that are a popular sport fish, and are often raised as fingerlings for stocking. However, the production of Walleye as a food fish is still limited in the United States due to a reliance on pond rearing and live feed during the larval stage, which lead to a limited production season [27]. Walleye is an altricial species, and although they have shown some acceptance of formulated feeds [27], an optimal larval feed for this species has not yet been developed. Production of a formulated dry feed for Walleye larvae could help increase intensive Walleye production by reducing live feed utilization and the need for outdoor ponds. Nile tilapia (*Oreochromis niloticus*) was used as a non-species-specific muscle and enzyme source because it is a species with distinctly different digestive and behavioral characteristics. Tilapia are tropical omnivores that have small stomachs [28]. In addition, Tilapia are a very popular aquaculture species, and their endogenous digestive enzymes have been extracted and used in previous in vitro hydrolysis studies [21].

## 2. Materials and Methods

### 2.1. Experimental Conditions

The feeding trial was conducted at the Center for Fisheries, Aquaculture, and Aquatic Sciences at Southern Illinois University-Carbondale (SIUC), IL. The experiment was conducted in strict accordance with the recommendations of the Guide for the Care and Use of Laboratory Animals of SIUC. All protocols performed were approved by SIUC Institutional Animal Care and Use (protocol #22–019). A semi-recirculated system supplied with groundwater was used, equipped with 30 black tanks (280 L), an established biofilter, and two mechanical (sand) filters (Pentair, Minneapolis, MN, USA). The photoperiod was maintained with overhead light strips and consisted of 24 h of dimmed light, with light intensity increased during feedings and cleanings. Tank conditions during the study were 20.38 °C (±2.03), with a pH of 7.40 (±0.55) and a constant inflow (100 mL/min) of system water. Salinity was maintained at 1–3 ppt throughout the study to prolong the viability of the live feed [29]. Each tank was equipped with a sprinkler to break the water surface and allow Walleye larvae to inflate their swim bladders [30]. Additionally, clay was added to the system each morning to increase the turbidity of the water. Four cups of very fine ball clay (OM-4) were mixed with system water and added to the system daily. This is common practice for rearing larval Walleye and is done to reduce cannibalism and clinging behavior in the tanks [31]. The sand filters were backflushed twice daily to prevent clay buildup from affecting the efficiency of the filtration system. 

### 2.2. Muscle Hydrolysis

The hydrolysis method for this study was based on the in vitro method described in Kwasek et al. [22], with some modifications. Eight different protein sources were produced, with one unhydrolyzed and one hydrolyzed protein obtained from each of the following combinations:

Walleye muscle with Walleye endogenous digestive enzymes;

Walleye muscle with Tilapia endogenous digestive enzymes;

Tilapia muscle with Walleye endogenous digestive enzymes;

Tilapia muscle with Tilapia endogenous digestive enzymes.

Both fresh and frozen muscles (50/50) and digestive tracts were used in this study. Both fresh and frozen muscle have been used to produce hydrolysates in the past [22,23,32]. Additionally, the use of frozen digestive tracts to obtain hydrolysates is supported by results obtained by Solovyev and Gisbert [33], which showed no significant decrease in the activity of digestive enzymes from digestive tracts that were frozen and stored for less than 270 days. The frozen digestive tracts utilized in this study were only stored for 60–70 days. The fresh muscles and digestive tracts were harvested from adult Walleye and Tilapia that were kept at 22 °C and 25 °C, respectively. On the morning of harvest, the fish were fed to satiation and euthanized 2 h after feeding with an excess of tricaine methanesulfonate (MS-222) (#E10521, Sigma-Aldrich, St. Louis, MO, USA) at a dose of 0.4 mg/mL. The muscle of each fish was isolated by removing the head and tail, and the digestive tract was dissected out and placed on ice. The obtained muscle was ground with a meat grinder (General Food Service, Weston, FL, USA) three times. Deionized water was added to the ground muscle (1:2 water:muscle), and the solution was homogenized (PowerGen 1000, Fisher Scientific, Waltham, MA, USA) at high speed for 10 min. The homogenized muscle was transferred to 12 L containers, placed in a water bath at 25 °C (for hydrolysis with Tilapia enzymes) or 22 °C (for hydrolysis with Walleye enzymes), diluted with deionized water (1:2), and stirred with an overhead stirrer (VWR VOS 16, VWR, Radnor, PA, USA) for the duration of hydrolysis. The digestive tracts were diluted with deionized water and homogenized under the same conditions as the muscle. The digestive tract solution was additionally centrifuged (1500× *g* for 10 min at 4 °C) to separate the solid mass (including undigested feed, fat, and tissues) and recover the supernatant. The recovered supernatant was then immediately added to the homogenized muscle (3:1 muscle:supernatant) to begin hydrolysis. The hydrolyzed products were continuously stirred for 3 h and the pH was adjusted to mimic digestion. The pH was maintained at 3–4 for the first hour to mimic gastric digestion and then increased to 7–9 for 2 h to mimic intestinal digestion [22]. After hydrolysis, the solution was brought to 95 °C for 15 min to stop enzymatic activity. The unhydrolyzed muscle products were brought to 95 °C immediately after mixing to prevent any hydrolysis. Although this product is not a fully unhydrolyzed protein, since it was mixed with digestive enzymes for a short time, it is referred to as unhydrolyzed to distinguish it from the products that underwent the incubated hydrolysis process. In addition to stopping the enzymatic activity, the 95 °C heat treatment applied to the protein products served to deactivate potential pathogens in the solution and prevent transfer of diseases from the adult fish to the larvae [34]. All products were frozen at –20 °C and then freeze-dried to remove moisture. After freeze-drying, lipids were extracted from each muscle product by the Soxhlet method using ethyl ether as the solvent [35]. Proteomic analysis of both unhydrolyzed and hydrolyzed products was performed at Ohio State University, Columbus, OH, USA.

### 2.3. Diets

Four diets were formulated to be isonitrogenous and isolipidic and to meet the essential nutrient requirements of Walleye larvae or closely related species [36]. They were formulated to contain 60% crude protein and 15% crude lipids. The formulations are listed in Table 1. The four feeds corresponded to the combination of muscles and enzymes used to produce the unhydrolyzed and hydrolyzed proteins:

W-W: Walleye muscle with Walleye endogenous digestive enzymes;

W-T: Walleye muscle with Tilapia endogenous digestive enzymes;

T-W: Tilapia muscle with Walleye endogenous digestive enzymes;

T-T: Tilapia muscle with Tilapia endogenous digestive enzymes.

The formulations for these experimental diets were based on a 50% replacement of unhydrolyzed protein with protein hydrolysates, similar to the formulations of Kwasek et al. [22]. 

### 2.4. Diet Analysis

The analyzed compositions of the formulated diets are presented in Table 2. Crude protein, crude lipid, moisture, and ash were quantified to obtain the proximate composition of the diets. Ash content in the diets was analyzed by combustion (550 °C for 5 h) in a muffle furnace (Lindberg Blue M, MA, USA). A Leco nitrogen analyzer (Model FP-628, Leco Corporation, St. Joseph, MO, USA) was used to assess crude protein (N × 6.25). Crude lipids were extracted with chloroform–methanol (2:1, *v*/*v*), as described by Folch et al. [37]. The amino acid composition of each diet was analyzed utilizing the Association of Official Analytical Chemists, International (AOAC) Official Method 999.13. All dietary samples were analyzed in technical triplicates.

### 2.5. Experimental Design

At 5 days post hatch (dph), Walleye larvae obtained from LaSalle Fish Hatchery (Illinois Department of Natural Resources, Marseilles, IL, USA) were randomly distributed to 18 tanks (280 L) containing ~9 fish/L. The feeding trial began at 6 dph. In this study, there were 6 treatment groups, each with 3 replicate tanks. The first 4 groups (W-W, W-T, T-W, and T-T), were fed one of the manufactured formulated diets and received the same-named diet for the duration of the feeding experiment. In addition, there was a live feed reference group that received only *Artemia franciscana* nauplii (Artemia) throughout the study, and a dry feed reference group that received a commercial starter feed (Otohime, Marubeni Nisshin Feed Co., Tokyo, Japan) (Com). 

All groups except for the Artemia group were fed a combination of *Artemia franciscana* nauplii and dry feed for the first 4 days of the experiment (6–9 dph) to ensure their survival, and then switched to dry feed (<250 µm powder) for the rest of the experiment (10–26 dph). The experiment was conducted until the fish fully metamorphosed into the juvenile stage (6–26 dph). Fish were fed in excess and, to ensure constant feed availability, were fed hourly from 08:00 to 16:00. Due to its high viscosity, the dry feed was added to the surface of the tanks through a sieve (250 µm) to avoid the formation of clumps and ensure the correct particle size corresponding to the larval gape size. The tanks were siphoned twice a day to prevent deterioration of water quality due to the accumulation of waste and uneaten feed. Approximately 2.5% of tank water was removed during each siphoning event. 

### 2.6. Sampling and Measuring

At the end of the study (26 dph), a subsample of 100 fish were collected from each tank and weighed together. The 100 fish from each tank were also visually assessed for common deformities seen in larval fish. These were tail- and head/jaw deformities, along with scoliosis and lordosis. The assessment of these deformities are often utilized as indicators of nutritional deficiencies in larval fish [38]. 

In addition to the fish collected for weight and deformity analysis, samples for FAA and PepT1 analyses were taken from each tank at the conclusion of the study. For gut PepT1 expression analyses, 3 fish from each tank were euthanized with an overdose of MS-222 and stored in RNAlater (Invitrogen by Fisher Scientific, Waltham, WA, USA). Sampling for PepT1 analysis was conducted 2 and 24 h after the final feeding, to assess PepT1 expression under postprandial and starvation states, respectively. The same timepoints were used to collect samples for FAA analysis, with the 2 and 24 h samples representing the postprandial and basal FAA levels, respectively. For each set of samples for FAA analyses, 3 fish from each tank were anesthetized with MS-222, euthanized in liquid nitrogen [39] and stored at –80 °C. 

### 2.7. PepT1 Analysis

Gene expression was analyzed by quantitative real-time polymerase chain reaction (qRT-PCR) as described in Terova et al. [40]. As the sequence of the PepT1 gene in Walleye was not available in the GenBank database, PepT1 was molecularly cloned and sequenced prior to transcript quantification in the Walleye intestine. 

Primers for amplification of a partial cDNA sequence of the PepT1 gene in Walleye were designed based on the sequence of Pikeperch (*Sander lucioperca*) (GenBank Acc. n° XM_031291729.2), Yellow perch (*Perca flavescens*) (Acc. n° GQ906471.2), and European sea bass (*Dicentrarchus labrax*) (Acc. N. FJ237043.2). A strategy based on regions of strong nucleotide conservation was used to design the primers. The sequences of the primers were: 5′-TCTTCTACCTGTCCATCAATGCT-3′ (Tm 59 °C) for the forward primer and 5′-TTCTCCTCAGCCCAGTCCAT-3′ (Tm 60 °C) for the reverse primer. 

For PCR amplification, an aliquot of Walleye intestinal cDNA was amplified using Taq PLATINUM SuperFi DNA Polymerase (Invitrogen by Fisher Scientific, Waltham, WA, USA) and the designed primers. The PCR thermal cycling conditions were as follows: initial denaturation at 98 °C for 30 s, followed by 35 cycles at 98 °C for 10 s, 55 °C for 10 s, 72 °C for 30 s, and a final elongation step at 72 °C for 5 min. To check the amplicon length, the PCR amplification product was loaded onto a 2% agarose gel containing ethidium bromide in TAE 1X buffer. The band of interest was extracted from the gel and purified using the NucleoSpin^®^ Gel, and PCR Clean-up Kit (Macherey-Nagel, Germany). DNA (insert of interest) was then ligated directly to the T-tailed plasmid vector pGEM^®^-T Easy (Promega Milan, Italy) and sequenced in both directions (T7 and SP6). Alignment using Clustal Omega software of the partial PepT1 cDNA sequence from Walleye with PepT1 from Pikeperch, Yellow perch, and European sea bass showed a high degree of similarity among the four fish species.

The final volume of each RT-qPCR sample was 20 µL, composed of 7 µL of Walleye cDNA (100 ng), 6 µL of iTaq Universal Probes Supermix (Bio-Rad, Milan, Italy), and 500 nM of each primer. The RT-qPCR was performed according to the manufacturer’s instructions on the CFX96 RT-PCR instrument (Bio-Rad, Milan, Italy). The thermal reaction conditions were as follows: 95 °C for 30 s, followed by 40 cycles of 95 °C for 10 s, and 60 °C for 30 s. A negative control was used in each assay, and consisted of nuclease-free water instead of the cDNA template. Relative expression levels were calculated using the 2^−∆∆CT^ method, with *β-actin* as the housekeeping gene (HK). Technical duplicates were analyzed for each sample with both the HK and target gene. With respect to the HK gene, three constitutive genes (*α-tubulin*, *eEF1a1*, and *β-actin*) were tested to determine the most stable one as a means of normalizing the relative quantification of PepT1 expression. The primers used for the HK genes were based on the sequences of each gene in Walleye available in the GenBank database.

The accession numbers of the sequences used to design the primers for HK genes are: MK860171.1 for *eEF1a1*, MK860170.1 for *α-tubulin*, and DQ231555.1 for *β-actin*. Primers were designed according to the instructions in the kit Probes Supermix. The melting temperature of the primers was 60 °C, whereas the melting temperature of the probes was at least 70 °C. The open-source Primer3 program with default settings was used to design the primers and probes. To achieve the best RT-qPCR efficiency, the amplicon length for each primer pair was set between 70 and 150 bp. The primers used for amplification of the target and HK genes are listed in Table 3.

Using CFX Maestro™ software (Bio-Rad, Milan, Italy), we were able to select the best reference gene and analyze its stability using the reference gene selection tool (CFX Maestro™ Software User Guide version 1.1). After this analysis, the software indicated that both *β-actin* and *eEF1α1* are the ideal HKs based on the average M value (M = 0.469) and stability value (0.758) (Supplementary Figure S1).

The efficiency of the amplification reaction was tested for each primer pair using CFX Maestro™ software. The amplification efficiency of each reaction was calculated as follows: Exponential Amplification = 10 (–1/slope). A standard curve was used to assess amplification efficiency. For this purpose, serial dilutions of walleye cDNA were prepared and a standard curve was generated for each primer pair. Typically, the desired amplification efficiencies range from 92% to 117%. In our case, each primer set showed an amplification efficiency of more than 80% (Supplementary Figure S2).

### 2.8. Free Amino Acid Analysis

Free amino acid analysis of the Walleye muscle was conducted using the method described in Kwasek et al. [22]. The muscle was dissected from the whole-body samples from each tank and combined to produce one sample per tank. The samples were homogenized with 0.1 M/L HCl at 1:9 (*w*/*v*). To obtain the supernatant, the homogenized muscle was centrifuged (12,000× g at 4 °C for 15 min), and the resulting supernatant was filtered (Milipore, 10-kDa cutoff at 15,000× *g*, 4 °C, 30 min) and diluted with 0.1 M/L HCl (1:19 *v*/*v*) containing norvaline and sarcosine (40 μM/L) as internal standards. External standards (Sigma acidic/neutral and basic amino acids) were produced, as were blank samples (0.1 M/L HCl + 40 μM/L norvaline and sarcosine). Glutamine in 0.1 M/L HCL was added to the basic amino acid standard as an external standard. Quantification of FAA was conducted with the Shimadzu Prominence Nexera—i LC-2040C Plus (Shimadzu, Japan) according to Shimadzu protocol No. L529, with modifications. Free amino acid concentrations (expressed as μM/g wet body weight) were calculated in LabSolutions software version 5.92 (Shimadzu, Japan) using internal and external standards.

### 2.9. SDS-PAGE

Sodium dodecyl sulfate-polyacrylamide gel electrophoresis (SDS-PAGE) was performed to visualize the molecular weight profile of the protein produced for this study. The electrophoretic analysis was conducted according to the method described by Updike et al. [41], with modifications. Samples were homogenized in dissociation buffer (8M urea, 2M thiourea, 60 mM Tris buffer, pH 6.8, containing 3% SDS, 350mM DDT, and 0.002% bromophenol blue) and then further diluted with dissociation buffer as needed. A discontinuous, reducing 12.5% T, SDS polyacrylamide gel was used, with a 3% T stacking gel. Sample dilutions (10 mg/mL) were loaded (~ 10 μL) into each lane of the stacking gel. The proteins were resolved at 150 V/cm until the dye front reached the bottom of the gel. Coomassie Brilliant Blue dye (40% methanol, 5% acetic acid, 0.04% Coomassie Brilliant Blue G-250) was used to stain the gel, followed by a destaining step with 10% acetic acid. The gel was then scanned on an AZURE c600 scanner (Azure Biosystems, Dublin, CA, USA).

### 2.10. Statistical Analysis

Statistical analysis was run using R version 3.5.2 (Boston, MA, USA). Growth performance, survival, skeletal deformity, and PepT1 expression data collected in this study were analyzed using the one-way ANOVA and a Tukey test to detect significant differences between groups. Differences between groups were considered significant at *p* < 0.05. Free amino acid data was analyzed using a one-way ANOVA, and differences between groups were tested using an LSD test. Differences between groups were considered significant at *p* < 0.05. Two-way ANOVAs were performed for the measured parameters between the hydrolysate-based groups (W-W, W-T, T-W, T-T) to evaluate interactions and main effects of muscle and enzyme source. Effects were considered significant at *p* < 0.05.

## 3. Results

### 3.1. SDS-PAGE

**Figure 1:**
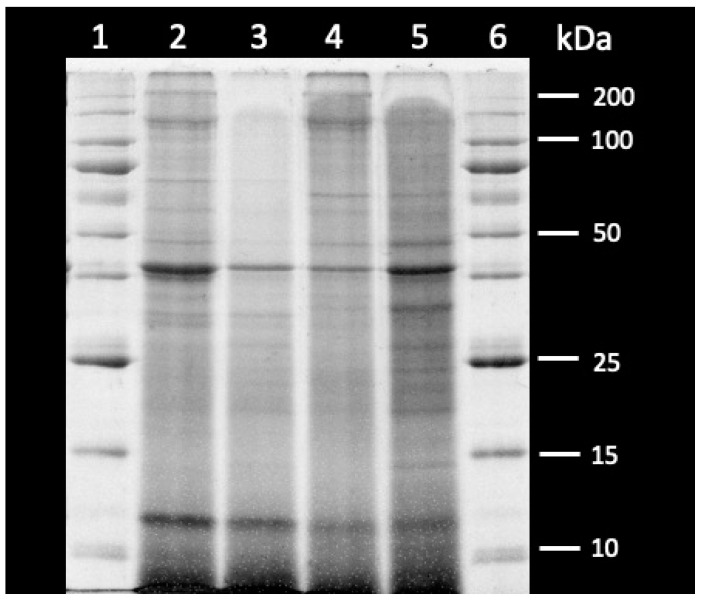
Representative reducing 12.5% T SDS-PAGE of dietary protein profile of experimental diets, represented by a 50:50 mix of the hydrolysate and unhydrolyzed protein produced with the corresponding muscle and enzymes. Lanes: (1) broad range molecular weight standard (200–10 kDa); (2) W-W; (3) W-T; (4) T-W; (5) T-T; (6) broad range molecular weight standard (10–200 kDa). W-W: Walleye muscle with Walleye enzymes; W-T: Walleye muscle with Tilapia enzymes; T-W: Tilapia muscle with Walleye enzymes; T-T: Tilapia muscle with Tilapia enzymes.

Figure 1 shows the representative dietary protein profile of each experimental diet produced for this study on a 12.5% SDS-PAGE gel. The product added into each lane contained 50% of the unhydrolyzed and 50% of the hydrolyzed protein from the corresponding muscle and enzyme combination (Lane 2: W-W; Lane 3: W-T; Lane 4: T-W; Lane 5: T-T). As neither the unhydrolyzed nor the hydrolyzed protein from each combination was solely used in a diet, the 50/50 combination of each product best represents the molecular weight profile of the dietary protein for each diet. The method used to produce the unhydrolyzed and hydrolyzed products has been described and reported previously [22,23].

The results of the SDS-PAGE gel show the molecular weight profiles of the dietary protein across the four muscle and enzyme combinations. Each lane containing the samples shows significant staining below 15 kDa, representing the presence of oligopeptides and possibly of single peptides produced from the hydrolysis of the muscle proteins. One observable difference between the molecular weight profiles of the diets is a reduced appearance of larger polypeptides and unhydrolyzed proteins (>50 kDa) in the W-T sample (Lane 3). In the case of the W-W (Lane 2), T-W (Lane 4), and T-T (Lane 5) diets, there is strong banding, which signifies the presence of these larger proteins at a range of molecular weights between 50 and 200 kDa.

### 3.2. Growth, Survival, and Skeletal Deformities

**Figure 2:**
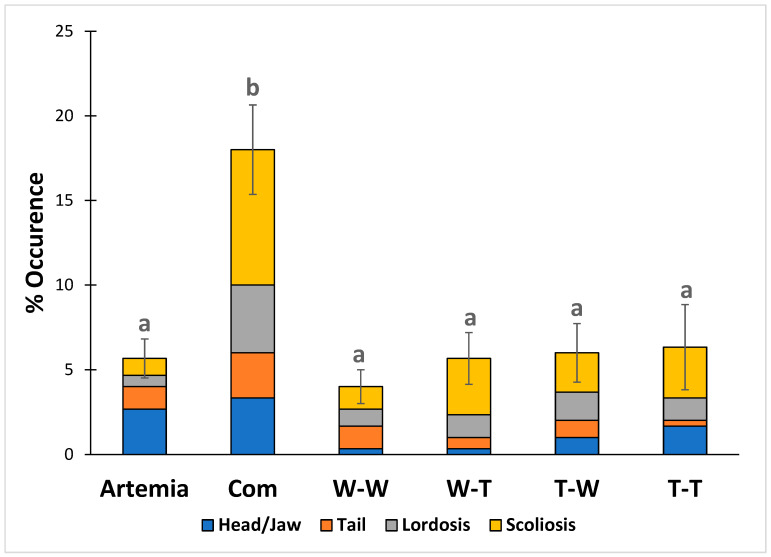
Diet effect on the occurrence of skeletal deformities (Lordosis, scoliosis, and head/jaw and tail deformities). Values are presented as means (±S.D.) for total deformities. Different letters indicate statistical significance between groups. The significance was determined using a one-way ANOVA and a Tukey test at *p* < 0.05.

Based on the one-way ANOVA and the Tukey test, the W-W group had a significantly higher average weight than all other groups at the end of the study. No significant differences in average weight were found between it and the other groups. During the study, the Artemia and Com groups had significantly higher survival rates than the four groups fed hydrolysate-based diets. No significant differences in survival were found among the groups fed hydrolysate-based diets, regardless of muscle or enzyme source. The results for growth and survival are shown in Table 4. In addition, the Com group had a significantly higher incidence of skeletal deformities compared to all other groups. No significant differences were observed among the other five groups. The results for skeletal deformities are shown in Figure 2.

The effects of muscle and enzyme source on growth, survival, and skeletal deformities were evaluated using two-way ANOVAs for the groups that received formulated diets from the different muscle and enzyme combinations. The results of the two-way ANOVAs are shown in Table 5. The interaction between muscle and enzymes was significant for larval growth. No significant interaction effect on survival was observed, but muscle source had a significant main effect, and the groups fed Walleye muscle-based diets had numerically lower survival. No interactions or main effects on the occurrence of skeletal deformities were observed in this study.

### 3.3. PepT1 Expression

**Figure 3:**
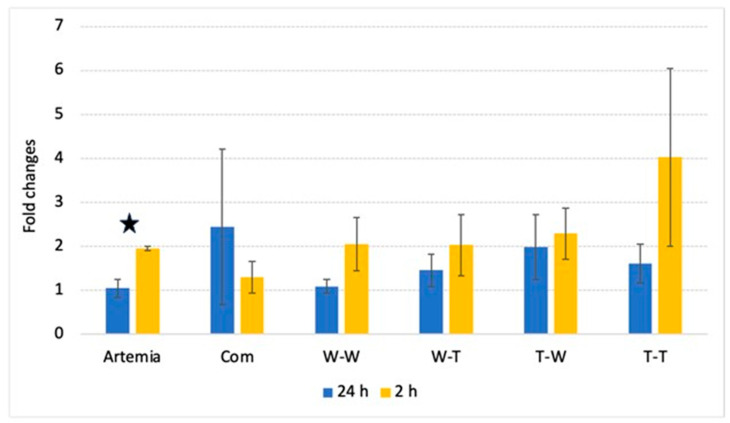
Results of qRT-PCR analysis on PepT1 expression. Values are presented as mean fold change and error bars represent standard error. Star indicate statistical significance (*p* < 0.05) between 24 h and 2 h expression.

There were no significant differences in PepT1 gene expression among groups 2 h and 24 h after feeding. However, PepT1 gene expression was significantly higher in the Artemia group 2 h after feeding than 24 h after. The results for PepT1 expression are shown in Figure 3.

The effects of muscle and enzyme source on postprandial PepT1 expression were examined using two-way ANOVAs for the groups that received formulated diets from the different muscle and enzyme combinations. The results of the two-way ANOVAs are shown in Table 5. In this study, no significant interactions or main effects were observed on the expression of PepT1 2 h after feeding.

### 3.4. Muscle Free Amino Acid Composition

**Figure 4:**
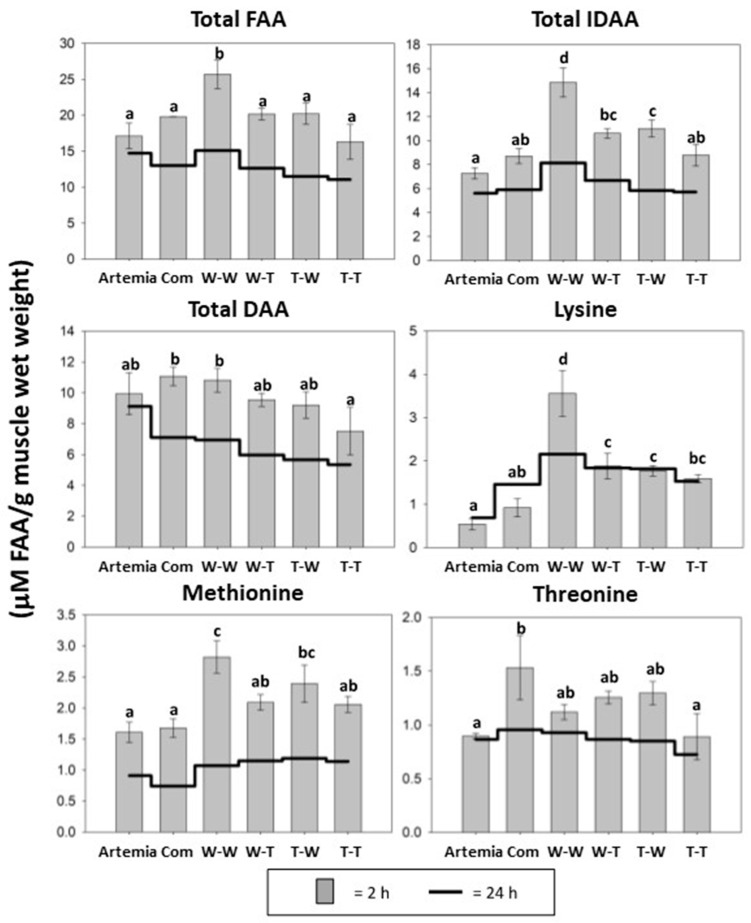
Muscle FAA levels in Walleye after 3 weeks of feeding. The gray bars represent FAA levels 2 h after feeding. The black line indicates FAA physiological baseline (24 h after feeding). Different letters indicate statistical difference at *p* < 0.05. Indispensable amino acids (IDAAs) = Ile, Leu, Lys, Met, Phe, Thr, Trp, Val, Arg, and His. Dispensable amino acids (DAAs) = Ala, Asp, Asn, Glu, Gln, Gly, Pro, Ser, and Tyr.

The W-W group had significantly higher concentrations of total FAA and total indispensable amino acids (IDAA) compared with all other groups (Figure 4). In addition, the T-W group had significantly higher total IDAA content than the Artemia, Com, and T-T groups, while the content in the W-T group was significantly higher than in the Artemia group. The total dispensable amino acid (DAA) content was significantly higher in the Com and W-W groups than in the T-T group. The effects of muscle and enzyme source on the postprandial muscle FAA pool were assessed with two-way ANOVAs for the groups that received formulated diets from the different muscle and enzyme combinations. The results of the two-way ANOVAs are shown in Table 5. There was no significant interaction effect on 2 h postprandial total FAA, total IDAA, or total DAA levels in muscle, but both muscle and enzyme source had significant main effects on these three parameters.

Among the individual amino acids, only free aspartic acid concentrations were not significantly different between the groups. The level of free glutamic acid and free threonine in muscle was significantly higher in the Com group compared to the T-T group, while the level of free threonine was also significantly higher than in the Artemia group. The content of free asparagine was significantly higher in the Artemia group than in all other groups except the Com group. In addition, the Artemia group had a significantly higher concentration of free serine than the W-W, T-W, and T-T groups. The free glutamine content was significantly lower in the T-T group than in the Com, W-W, and T-W groups. The W-W group had a significantly higher level of free histidine, glycine, and arginine in the muscle compared to all other groups, and a significantly higher level of free tyrosine than the Artemia and Com groups. In addition, the W-T and T-W groups had significantly higher levels of free arginine and lysine than the Artemia group. The Com group had significantly higher levels of free alanine in muscle than all other groups except the Artemia group. The free methionine content was significantly lower in the Artemia and Com groups than in the W-W and T-W groups, while the W-W concentration was significantly higher than in all other groups except the T-W group. Free valine concentration in the W-T and T-T groups was significantly higher than in the Artemia and Com groups, but significantly lower than in the W-W and T-W groups. The T-T group had significantly lower free tryptophan content than the W-W and W-T groups. The W-T groups had significantly higher free phenylalanine content than the Artemia and Com groups. Free isoleucine and leucine concentrations were significantly higher in the W-T group compared to all groups except the W-W group, while the T-T group also had significantly lower free isoleucine content compared to the Com and W-W groups. The Artemia group had a significantly lower concentration of free proline compared to all groups except the Com and T-T groups. Postprandial results for FAAs analyzed are shown in Table 6 (2 h) and Table 7 (24 h).

## 4. Discussion

Previous studies have compared different muscle sources for fish protein hydrolysate production [6,42,43]. In Asian seabass (*Lates calcarifer*), both fish muscle and squid mantle were hydrolyzed by Alcalase^®^ and used to replace 50% of the fishmeal in the respective diets [6]. The fish fed the fish protein hydrolysate recorded a significantly higher weight gain than the fish fed squid mantle hydrolysate [6]. Khosravi et al. [42] found that the addition of a krill hydrolysate significantly increased the growth of both Red sea bream (*Pagrus major*) and Olive flounder (*Paralichthys olivaceus*), while a tuna hydrolysate had no significant effect on growth in either species. Different enzyme sources for fish protein hydrolysate production have also been tested, both commercial [15,17,44] and endogenous [16,21,22,23]. Kristinsson and Rasco [15] suggested that endogenous digestive enzymes are more ideal for the production of protein hydrolysates. Direct comparisons between commercial and endogenous enzymes showed that extracted enzymes from fish viscera hydrolyze proteins more efficiently and achieve a higher degree of hydrolysis than the commercial enzymes used [21,44].

The present study compared two different fish species as sources of muscle and endogenous digestive enzymes to produce an optimal fish protein hydrolysate for Walleye larvae. The results of this study showed a significant increase in final weight in the W-W group compared to all other groups. Fish in the W-W group weighed 30% more than the next heaviest group (W-T) at the end of the study. The significant increase in growth compared to the Artemia and Com groups should be emphasized. These groups were used as a reference because they represent two current feeding systems in rearing Walleye larvae: either a commercial starter feed (Com) or live feed (Artemia) are used until the fish metamorphose into juveniles [45,46]. Our results show that there was a significant interaction effect between the muscle and enzyme sources on growth, and the species-specific combination resulted in a protein hydrolysate that promoted larval growth that was higher than with current feeding regimes for Walleye larvae replicated in this study.

The use of species-specific muscle as a protein source is based on the long-held theory that adult fish muscle contains the ideal amino acid profile for larvae of the same species [47,48]. Therefore, the use of species-specific muscle in larval diets seems to be the most optimal protein source to meet the amino acid requirements for Walleye larvae growth and development. This study served as a practical proof of concept for this theory. Research based on this concept is important as a means of furthering the understanding of nutritional requirements for larval fish, and the specific IDAA requirements for each species. Given the natural cannibalistic behavior observed in Walleye [49] and in other cultured fish species [50,51,52], the use of same-species muscle as a protein source seems to fall within ethical guidelines for the purpose of this research. The use of hydrolyzed muscle from the same species has been tested previously [14,23]. Sandbakken et al. [14] found that partial replacement of fishmeal with a salmon hydrolysate increased the specific growth rate of Atlantic salmon (*Salmo salar*) during the first 25 days of feeding. However, the salmon hydrolysate used in that study was compared to a fishmeal derived from multiple marine species, so there was no comparison between the salmon hydrolysate and a hydrolysate produced from another single species. The results of this study show that species-specific muscle leads to increased weight gain in Walleye larvae, and hydrolysis by species-specific enzymes seems to have made the additional contribution to this significantly improved growth performance.

The use of species-specific enzymes to produce the hydrolysate was carried out to break down the protein in the same way that living fish would, but with an in vitro approach. Mimicking the in vivo digestion of the dietary protein for the larval fish served to compensate for the lack of mature enzymatic activity in the larval fish [53]. The concept of mimicking in vivo digestion has been used in previous studies to evaluate the digestibility of raw materials for various fish species [54,55,56]. Hansen et al. [54] tested this concept, specifically for predicting the digestibility of larval fish feeds using the pH-stat method. For this method, a constant pH is maintained during the hydrolysis reaction by titration and the degree of hydrolysis is measured based on the amount of basic solution added during the reaction. Commercial enzymes and endogenous enzymes from Atlantic cod (*Gadus morhua*) were utilized for that study, and only the endogenous enzymes were able to identify significant differences in digestibility between the two diets tested [54]. Ultimately, the diet identified as more digestible by the cod enzyme method significantly increased growth of the cod larvae [54]. Previous studies have shown that there is a significant relationship between apparent in vivo protein digestibility and analyzed in vitro digestibility of protein sources using species-specific endogenous enzymes [55,56]. The end products of these in vitro digestibility methods are hydrolyzed proteins that theoretically represent a dietary protein profile tailored to the digestive physiology of each specific species.

In previous studies, in vivo digestion was mimicked in processes similar to in vitro digestibility tests, and the hydrolyzed protein produced was used in formulated dry diets for larval fish [22,23]. Kwasek et al. [22] found that the use of a muscle hydrolysate from Bighead carp (*Hypophthalmichthys nobilis)* produced with endogenous digestive enzymes from adult Largemouth bass (*Micropterus salmoides*) increased the growth performance of larval Largemouth bass. Molinari et al. [23] tested the use of both muscle and endogenous digestive enzymes from adult Largemouth bass to produce hydrolysates and found that the growth of larval Largemouth bass during the first week of feeding was significantly higher than that of larvae fed a diet based on unhydrolyzed protein from the same source. While this earlier study used species-specific muscle and enzymes, no comparison was made with muscle and enzymes from other species. Building on the results observed in those studies, the results of the present study demonstrate that in a direct comparison, the combination of species-specific muscle and enzymes yields the more optimal protein hydrolysate for larval fish growth compared to muscle and enzymes from other fish species.

The mechanism behind the increase in growth in the W-W group could be a higher availability of amino acids in the diet and, thus, increased absorption success. Postprandial FAA results showed that Walleye muscle hydrolyzed with Walleye endogenous digestive enzymes significantly increased the level of absorbed amino acids 2 h after feeding. The W-W group had significantly higher levels of total FAA, total IDAA, and free lysine in the muscle FAA pool than all other groups in the study. The increase in postprandial FAA likely reflects an increase in dietary amino acids available for protein synthesis. Protein synthesis in growing fish depends on a balanced FAA pool to synthesize protein and store it as new tissue [36]. Increased dietary IDAA absorption into the FAA pool is particularly critical because these amino acids cannot be synthesized in the body. Expression of the intestinal di-/tripeptide transporter PepT1 was analyzed to gain insight into the efficiency of protein absorption from the different diets [57,58], but no significant differences in postprandial expression of PepT1 were observed between the groups. The results of the two-way ANOVA show that neither muscle nor enzyme source had a significant effect on postprandial expression of PepT1, suggesting that the absorption levels of di-/tripeptides were similar across all the hydrolysate-based diets. Therefore, the significant differences in postprandial FAA composition of muscles in the W-W group could be due to dietary protein being absorbed in the intestine as FAA and not as di-/tripeptides. The use of species-specific sources for the preparation of fish protein hydrolysates was done to optimize the absorption and utilization of dietary protein by providing the ideal amino acid composition with muscle specifically digested for optimal absorption by endogenous enzymes. While no interaction effect was observed between muscle and enzyme source, there were significant main effects of each source that appeared to be additive and contributed to the significant increase in FAA absorption in the W-W group.

In addition to the positive effects on growth, our study found that the addition of protein hydrolysates significantly reduced the incidence of skeletal deformities, regardless of muscle or enzyme source. This was an expected result, as it has been observed in many previous studies [22,23,59,60,61]. Skeletal deformities are detrimental to the aquaculture industry because deformed fish are often considered lower-grade products [62,63,64]. The occurrence of skeletal deformities has been attributed to many different nutritional deficiencies, including vitamin [65], phosphorous [66], magnesium [67], essential fatty acid [38], phospholipid [68], and amino acid deficiencies [63,69]. The relationship between dietary amino acid intake and skeletal deformities was of interest in this study because species-specific muscle was selected to provide fish larvae with the ideal amino acid profile. However, the muscle source did not significantly affect the occurrence of skeletal deformities, nor did the endogenous digestive enzyme source.

In contrast to the positive effects on skeletal development, dietary inclusion of each of the protein hydrolysates produced in this study significantly reduced survival compared to the Com and Artemia reference groups. The reduction in survival compared to the Artemia group was not solely due to the inclusion of protein hydrolysates, as high mortality was observed in previous studies attempting to replace live feeds with various dry feeds [70,71,72]. However, the lower survival rate in the hydrolysate groups compared to the Com group is noteworthy because the Com group received dry feeds in the same quantity and duration as the hydrolysate groups. Although the differences in nutritional and physical properties between the commercial and experimental diets do not allow for a direct comparison of survival rates, the lower survival rate may be a significant barrier to the use of hydrolysates in larval diets.

This reduction in survival has been observed in previous studies examining the inclusion of hydrolysates in larval feeds [5,6,23,73], and has been attributed to increased leaching of hydrolyzed protein [74,75,76]. Leaching significantly reduces the nutritional value of feeds due to the loss of water-soluble proteins [76]. Protein leaching rates increase with increasing levels of hydrolysis, suggesting that more leaching occurs in diets with high levels of hydrolysates than in those with unhydrolyzed dietary protein [6,73,74,75]. Given the 37% inclusion level of the hydrolysates in this study, which accounted for 50% of dietary protein, it is likely that significant leaching from each of our hydrolysate-based diets occurred, reducing the nutritional value of the feed particles suspended in the water column. Although feed intake in this study was monitored very closely and feed was added in excess and at short intervals to ensure a constant supply of fresh food, leaching from hydrolysate-based feeds has been found to occur rapidly [76], with leaching of protein from small feed particles reaching its maximum within 1–5 min [74]. Thus, it is feasible that some animals in the tanks that ingested the food particles more slowly and received feed that had experienced leaching of protein were more susceptible to mortality than the Com and Artemia reference groups. This lower survival highlights the importance of optimal physical properties of the feed to ensure that the fish receive the full nutritional profile of the diet. The use of binders such as guar gum and alginate can help increase pellet stability [36,77], reduce nutrient leaching, and further improve the future use of hydrolysate-based feeds in aquaculture.

## 5. Conclusion

Overall, the results of this study showed that there was a significant interaction effect between the muscle and endogenous digestive enzyme source used to produce protein hydrolysates, and that species-specific muscle and enzymes produced a more efficient hydrolysate for larval growth compared to other combinations of muscle and enzymes. The combination of muscle and endogenous digestive enzymes from the same species provided an optimal amino acid profile that was broken down for efficient uptake and utilization. This species-specific combination was able to significantly increase the postprandial total FAA and IDAA content in muscle, possibly leading to increased protein synthesis and the increased weight gain observed in the W-W group. Future research should focus on improving the physical properties of the formulated feeds, either through binders or encapsulation techniques. This should be done to reduce possible leaching of the hydrolyzed protein in the feed and improve survival of fish larvae receiving hydrolysate-based diets.

## Figures and Tables

**Table 1:** Diet formulations for Walleye feeding trial.

Ingredients (%)	Diet
Unhydrolyzed Protein ^a^	37.00
Protein Hydrolysate ^b^	37.00
Krill Meal ^c^	5.00
Fish oil ^d^	9.00
Lecithin ^e^	4.00
Mineral mix ^f^	3.00
Vitamin mix ^g^	3.00
CaHPO4	1.00
Taurine	1.00
Choline chloride	0.10
Vitamin C ^h^	0.05
Sum	100

^a^ Unhydrolyzed muscle protein that did not undergo incubated hydrolysis. ^b^ Hydrolyzed muscle protein produced with in vitro hydrolysis method. ^c^ Processed *Euphausia superba* (Florida Aqua Farms, Dade City, FL, USA). ^d^ Cod liver oil (MP Biomedicals, Solon, OH, USA). ^e^ Refined soy lecithin (MP Biomedicals, Solon, OH, USA). ^f^ Bernhart-Tomarelli mineral mix with 5 ppm selenium in a form of sodium selenite (Dyets, Bethlehem, PA, USA). ^g^ Custom Vitamin Mixture (mg/kg diet) Thiamin HCl, 6.84; Riboflavin, 7.2; Pyridoxine HCl, 10.29; Niacin, 16.35; D-Calcium Pantothenate, 75.84; Folic Acid, 1.89; D-Biotin, 0.24; Vitamin B12 (0.1%), 30; Vitamin A Palmitate (500,000 IU/g), 14.49; Vitamin D3 (400,000 IU/g), 12.39; Vitamin E Acetate (500 IU/g), 198; Menadione Sodium Bisulfite, 3.54; Inositol, 750 (Dyets, Bethlehem, PA, USA). ^h^ L-Ascorbyl-2-Polyphosphate (Argent Aquaculture, Redmond, WA, USA).

**Table 2:** Analyzed composition of formulated diets. Diets were analyzed in technical triplicates and the values are presented as means (±S.D.). Indispensable amino acids (IDAA) = Ile, Leu, Lys, Met, Phe, Thr, Trp, Val, Arg, and His. Dispensable amino acids (DAA) = Ala, Asp, Cys, Glu, Gly, Hyp, Pro, Ser, Tau and Tyr.

Analyzed Composition (g/100g) Dry Matter	W-W	W-T	T-W	T-T
Crude Protein (N × 6.25)	60.23 (±0.09)	60.20 (±0.15)	59.92 (±0.25)	59.93 (±0.05)
Crude Lipids	15.30 (±0.05)	15.47 (±0.02)	15.66 (±0.02)	15.08 (±0.08)
Ash	13.48 (±0.21)	13.45 (±0.09)	13.82 (±0.09)	13.74 (±0.45)
Alanine	3.70 (±0.02)	3.86 (±0.13)	3.73 (±0.01)	3.77 (±0.09)
Arginine	3.40 (±0.03)	3.08 (±0.12)	3.57 (±0.01)	3.17 (±0.09)
Aspartic Acid	5.58 (±0.02)	5.63 (±0.04)	5.30 (±0.07)	5.45 (±0.09)
Cysteine	0.59 (±0.02)	0.63 (±0.00)	0.53 (±0.01)	0.58 (±0.02)
Glutamic Acid	7.97 (±0.24)	7.81 (±0.10)	7.71 (±0.03)	7.97 (±0.09)
Glycine	3.86 (±0.04)	4.20 (±0.28)	4.51 (±0.00)	4.35 (±0.32)
Histidine	1.49 (±0.02)	1.43 (±0.03)	1.27 (±0.01)	1.36 (±0.03)
Hydroxyproline	0.60 (±0.02)	0.65 (±0.01)	1.13 (±0.11)	0.94 (±0.14)
Isoleucine	2.65 (±0.03)	2.66 (±0.04)	2.49 (±0.06)	2.59 (±0.08)
Leucine	4.31 (±0.03)	4.31 (±0.05)	4.07 (±0.02)	4.23 (±0.09)
Lysine	5.04 (±0.03)	4.61 (±0.07)	4.52 (±0.01)	4.28 (±0.08)
Methionine	1.69 (±0.01)	1.70 (±0.00)	1.45 (±0.03)	1.51 (±0.02)
Phenylalanine	2.32 (±0.02)	2.34 (±0.05)	2.17 (±0.03)	2.28 (±0.04)
Proline	2.34 (±0.03)	2.50 (±0.08)	2.70 (±0.04)	2.63 (±0.14)
Serine	2.26 (±0.11)	2.24 (±0.05)	2.02 (±0.06)	2.00 (±0.02)
Taurine	1.53 (±0.01)	1.54 (±0.03)	1.87 (±0.03)	1.86 (±0.13)
Threonine	2.51 (±0.00)	2.56 (±0.01)	2.41 (±0.03)	2.47 (±0.03)
Tryptophan	0.61 (±0.03)	0.64 (±0.03)	0.54 (±0.01)	0.61 (±0.03)
Tyrosine	1.79 (±0.01)	1.80 (±0.05)	1.62 (±0.11)	1.73 (±0.04)
Valine	2.83 (±0.04)	2.91 (±0.02)	2.73 (±0.04)	2.84 (±0.05)
IDAA	26.85 (±0.11)	26.24 (±0.36)	25.22 (±0.19)	25.34 (±0.34)
DAA	30.21 (±0.35)	30.87 (±0.44)	31.12 (±0.13)	31.29 (±0.50)
Total	57.06 (±0.24)	57.11 (±0.20)	56.33 (±0.27)	56.63 (±0.41)

**Table 3:** Primers used to amplify target genes.

Gene	Primer	Nucleotide Sequence (5′–3′)
PepT1	Forward	CACACCCAGCAGAAGTGCTACT
	Reverse	ACAATCAGAGCTACCACCATGAGA
	Probe	FAM-ACTGGCCTTTGGTGTCCCCGC-NFQ
*eEF1a1*	Forward	GGAAATCCGTCGTGGATATGTG
	Reverse	TGACCTGGGCGTTGAAGTTG
	Probe	FAM-CTGGCGACAGCAAGAACGACCCACC-NFQ
*α-tubulin*	Forward	ACCAACCTCAACAGGCTAATTGG
	Reverse	GAGGGCACCATCGAAACGA
	Probe	FAM-CAGATTGTGTCCTCCATCACTGCCTCCC-NFQ
*β-actin*	Forward	CCCTCTTCCAGCCTTCCTT
	Reverse	GTAGGTGGTCTCGTGGATTCC
	Probe	FAM-CCTCGGTATGGAGTCCTG-NFQ

**Table 4:** Diet effect on growth and survival. Values are presented as means (±S.D.). Superscript letters indicate statistical significance between groups. The significance was determined using a one-way ANOVA and a Tukey test at *p* < 0.05.

Group	Avg. Weight (mg)	Survival (%)
Artemia	61.01 ^a^ (±12.47)	53.63 ^b^ (±11.43)
Com	57.35 ^a^ (±6.18)	39.41 ^b^ (±1.39)
W-W	89.84 ^b^ (±6.09)	16.87 ^a^ (±5.71)
W-T	62.83 ^a^ (±4.03)	13.95 ^a^ (±2.41)
T-W	61.22 ^a^ (±9.61)	20.98 ^a^ (±3.16)
T-T	54.70 ^a^ (±8.62)	21.69 ^a^ (±4.80)

**Table 5:** Results of two-way ANOVA. In the presence of no significant (NS) interaction effect, the *p*-values for each main effect are presented. Effects were determined to be significant at *p* < 0.05 and are indicated with an asterisk *.

Parameter	Factor	*p*-Value
Avg. Weight	Muscle Source	-
	Enzyme Source	-
	Interaction *	0.0437
Survival	Muscle Source *	0.0344
	Enzyme Source	0.6553
	Interaction	NS
Total Occurrence of Skeletal Deformities	Muscle Source	0.2126
	Enzyme Source	0.3406
	Interaction	NS
Postprandial (2 h) PepT1 Expression	Muscle Source	0.3442
	Enzyme Source	0.4652
	Interaction	NS
Postprandial (2 h) Total FAA Level in Muscle	Muscle Source *	0.0013
	Enzyme Source *	0.0012
	Interaction	NS
Postprandial (2 h) Total IDAA Level in Muscle	Muscle Source *	0.0008
	Enzyme Source *	0.0003
	Interaction	NS
Postprandial (2 h) Total DAA Level in Muscle	Muscle Source *	0.0085
	Enzyme Source *	0.0243
	Interaction	NS

**Table 6:** Postprandial FAA compositon in muscle. The values presented are postprandial levels, 2 h after feeding. Values are presented as means (±S.D.). Superscript letters indicate statistical significance between groups. The significance was determined using a one-way ANOVA and an LSD test at *p* < 0.05. Indispensable amino acids (IDAAs) = Ile, Leu, Lys, Met, Phe, Thr, Trp, Val, Arg, and His. Dispensable amino acids (DAAs) = Ala, Asp, Asn, Glu, Gln, Gly, Pro, Ser, and Tyr. TFAA = total free amino acids. Asterisk * signifies non-detect of cysteine in samples.

Amino Acid (μM/g)	Artemia	Com	W-W	W-T	T-W	T-T
Aspartic Acid	1.00 (±0.16)	1.41 (±0.25)	1.03 (±0.10)	1.16 (±0.04)	1.09 (±0.14)	1.00 (±0.19)
Glutamic Acid	1.78 ^ab^ (±0.18)	2.14 ^b^ (±0.28)	1.84 ^ab^ (±0.15)	1.86 ^ab^ (±0.03)	1.80 ^ab^ (±0.19)	1.45 ^a^ (±0.21)
Asparagine	0.44 ^c^ (±0.12)	0.36 ^bc^ (±0.09)	0.20 ^ab^ (±0.01)	0.17 ^a^ (±0.02)	0.19 ^ab^ (±0.03)	0.15 ^a^ (±0.06)
Serine	1.55 ^b^ (±0.50)	1.20 ^ab^ (±0.33)	0.68 ^a^ (±0.04)	0.84 ^ab^ (±0.06)	0.70 ^a^ (±0.11)	0.53 ^a^ (±0.18)
Glutamine	0.50 ^ab^ (±0.02)	0.58 ^b^ (±0.04)	0.63 ^b^ (±0.03)	0.52 ^ab^ (±0.03)	0.59 ^b^ (±0.08)	0.38 ^a^ (±0.08)
Histidine	1.77 ^a^ (±0.48)	1.73 ^a^ (±0.28)	2.66^b^ (±0.04)	1.53 ^a^ (±0.11)	1.63 ^a^ (±0.28)	1.07 ^a^ (±0.31)
Glycine	1.96 ^a^ (±0.67)	1.75 ^a^ (±0.44)	3.36 ^b^ (±0.05)	1.95 ^a^ (±0.12)	1.91^a^ (±0.24)	1.86^a^ (±0.41)
Threonine	0.90 ^a^ (±0.03)	1.53 ^b^ (±0.30)	1.21 ^ab^ (±0.07)	1.26 ^ab^ (±0.06)	1.30 ^ab^ (±0.11)	0.89 ^a^ (±0.22)
Arginine	0.29 ^a^ (±0.03)	0.45 ^ab^ (±0.08)	1.04 ^c^ (±0.17)	0.53 ^b^ (±0.03)	0.63 ^b^ (±0.02)	0.44 ^ab^ (±0.08)
Alanine	1.80 ^bc^ (±0.51)	2.43 ^c^ (±0.27)	1.49 ^ab^ (±0.16)	1.64 ^ab^ (±0.17)	1.37 ^ab^ (±0.12)	0.89 ^a^ (±0.25)
Tyrosine	0.45 ^a^ (±0.02)	0.46 ^a^ (±0.08)	0.79 ^b^ (±0.15)	0.62 ^ab^ (±0.06)	0.55 ^ab^ (±0.03)	0.54 ^ab^ (±0.14)
Lysine	0.55 ^a^ (±0.14)	0.93 ^ab^ (±0.22)	3.57^d^ (±0.53)	1.88 ^c^ (±0.30)	1.77 ^c^ (±0.12)	1.59 ^bc^ (±0.09)
Methionine	1.61 ^a^ (±0.16)	1.68 ^a^ (±0.15)	2.83 ^c^ (±0.26)	2.10 ^ab^ (±0.12)	2.40 ^bc^ (±0.30)	2.06 ^ab^ (±0.13)
Valine	1.44 ^a^ (±0.07)	1.49 ^a^ (±0.09)	2.40 ^c^ (±0.07)	2.03 ^b^ (±0.10)	2.39 ^c^ (±0.12)	2.04 ^b^ (±0.11)
Cysteine * Not detected	-	-	-	-	-	-
Tryptophan	0.03 ^ab^ (±0.01)	0.02 ^ab^ (±0.01)	0.04 ^b^ (±0.00)	0.04 ^b^ (±0.00)	0.03 ^a b^ (±0.00)	0.02 ^a^ (±0.01)
Phenylalanine	0.19 ^a^ (±0.03)	0.17 ^a^ (±0.02)	0.25 ^ab^ (±0.03)	0.27 ^b^ (±0.02)	0.19 ^ab^ (±0.03)	0.20 ^ab^ (±0.04)
Isoleucine	0.18 ^ab^ (±0.02)	0.23 ^bc^ (±0.04)	0.28 ^cd^ (±0.03)	0.32 ^d^ (±0.00)	0.21 ^ac^ (±0.02)	0.14 ^a^ (±0.03)
Leucine	0.32 ^a^ (±0.07)	0.47 ^ab^ (±0.10)	0.59 ^bc^ (±0.05)	0.66 ^c^ (±0.00)	0.47 ^ab^ (±0.05)	0.34 ^a^ (±0.07)
Proline	0.46 ^a^ (±0.06)	0.74 ^ac^ (±0.11)	0.80 ^bc^ (±0.10)	0.80 ^b c^ (±0.02)	1.02 ^c^ (±0.19)	0.71 ^ab^ (±0.10)
IDAA	7.27 ^a^ (±0.47)	8.71 ^ab^ (±0.63)	14.87^d^ (±1.23)	10.61 ^bc^ (±0.41)	11.02 ^c^ (±0.72)	8.78 ^ab^ (±0.90)
DAA	9.94 ^ab^ (±1.36)	11.09 ^b^ (±0.60)	10.82 ^b^ (±0.77)	9.56 ^ab^ (±0.43)	9.20 ^ab^ (±0.87)	7.52 ^a^ (±1.56)
TFAA	17.21 ^a^ (±1.79)	19.80 ^a^ (±0.03)	25.69 ^b^ (±2.00)	20.17 ^a^ (±0.84)	20.22 ^a^ (±1.49)	16.30 ^a^ (±2.43)

**Table 7:** Basal FAA composition in muscle. The values presented are basal levels, 24 h after feeding. Values are presented as means (±S.D.). Indispensable amino acids (IDAAs) = Ile, Leu, Lys, Met, Phe, Thr, Trp, Val, Arg, and His. Dispensable amino acids (DAAs) = Ala, Asp, Asn, Glu, Gln, Gly, Pro, Ser, and Tyr. TFAA = total free amino acids. Asterisk * signifies non-detect of cysteine in samples.

Amino Acid (μM/g)	Artemia	Com	W-W	W-T	T-W	T-T
Aspartic Acid	0.68 (±0.02)	1.29 (±0.30)	0.75 (±0.06)	0.87 (±0.02)	0.74 (±0.02)	0.93 (±0.05)
Glutamic Acid	1.38 (±0.03)	1.74 (±0.26)	1.57 (±0.10)	1.53 (±0.10)	1.18 (±0.03)	1.36 (±0.05)
Asparagine	0.35 (±0.05)	0.15 (±0.04)	0.15 (±0.01)	0.11 (±0.01)	0.12 (±0.01)	0.11 (±0.01)
Serine	1.23 (±0.08)	0.57 (±0.09)	0.42 (±0.01)	0.39 (±0.02)	0.45 (±0.01)	0.39 (±0.04)
Glutamine	0.54 (±0.04)	0.40 (±0.06)	0.35 (±0.02)	0.40 (±0.04)	0.31 (±0.01)	0.31 (±0.00)
Histidine	1.52 (±0.12)	0.54 (±0.11)	1.26 (±0.22)	0.55 (±0.03)	0.51 (±0.03)	0.39 (±0.00)
Glycine	2.32 (±0.17)	0.76 (±0.18)	1.97 (±0.08)	1.04 (±0.01)	1.30 (±0.29)	1.04 (±0.06)
Threonine	0.87 (±0.03)	0.96 (±0.22)	0.93 (±0.09)	0.87 (±0.07)	0.85 (±0.08)	0.73 (±0.06)
Arginine	0.17 (±0.00)	0.22 (±0.02)	0.38 (±0.01)	0.30 (±0.04)	0.36 (±0.03)	0.22 (±0.05)
Alanine	1.66 (±0.08)	1.32 (±0.26)	0.83 (±0.10)	0.92 (±0.07)	0.87 (±0.03)	0.73 (±0.06)
Tyrosine	0.44 (±0.08)	0.40 (±0.04)	0.56 (±0.01)	0.47 (±0.02)	0.60 (±0.01)	0.28 (±0.26)
Lysine	0.69 (±0.12)	1.46 (±0.26)	2.16 (±0.06)	1.85 (±0.31)	1.82 (±0.40)	1.53 (±0.62)
Methionine	0.91 (±0.17)	0.74 (±0.06)	1.07 (±0.10)	1.15 (±0.25)	1.19 (±0.06)	1.14 (±0.07)
Valine	1.10 (±0.11)	1.45 (±0.06)	1.90 (±0.05)	1.56 (±0.39)	0.80 (±0.48)	1.17 (±0.02)
Cysteine * Not detected	-	-	-	-	-	-
Tryptophan	0.02 (±0.00)	0.03 (±0.00)	0.02 (±0.00)	0.03 (±0.02)	0.04 (±0.00)	0.06 (±0.01)
Phenylalanine	0.09 (±0.01)	0.14 (±0.01)	0.12 (±0.01)	0.10 (±0.01)	0.08 (±0.03)	0.12 (±0.02)
Isoleucine	0.08 (±0.01)	0.11 (±0.01)	0.09 (±0.01)	0.07 (±0.04)	0.02 (±0.00)	0.11 (±0.02)
Leucine	0.16 (±0.03)	0.25 (±0.01)	0.22 (±0.03)	0.19 (±0.10)	0.17 (±0.08)	0.24 (±0.01)
Proline	0.53 (±0.10)	0.49 (±0.09)	0.37 (±0.03)	0.22 (±0.03)	0.10 (±0.08)	0.20 (±0.04)
IDAA	5.61 (±0.30)	5.90 (±0.68)	8.14 (±0.16)	6.67 (±0.57)	5.84 (±0.92)	5.71 (±0.86)
DAA	9.12 (±0.41)	7.12 (±1.22)	6.95 (±0.14)	5.97 (±0.29)	5.66 (±0.37)	5.35 (±0.25)
TFAA	14.73 (±0.63)	13.02 (±1.84)	15.09 (±0.23)	12.64 (±0.74)	11.50 (±1.19)	11.06 (±1.09)

## Data Availability

The original contributions presented in the study are included in the article/supplementary material; further inquiries can be directed to the corresponding author.

## Supplementary Materials

The following supporting information can be downloaded at: https://www.mdpi.com/article/10.3390/ani14172493/s1, Figure S1: Results of stability analysis of the housekeeping genes *eEF1a1*, *α-tubulin*, and *β-actin.*; Figure S2: Results of amplification efficiency of each pair of primers for the target and housekeeping genes *eEF1a1*, *α-tubulin*, *β-actin*, and PepT1.
