# Supplementary material for: The Effect of the Species Source of Muscle and/or Digestive Enzymes on the Utilization of Fish Protein Hydrolysates as a Dietary Protein Source in First Feed for Larval Walleye (Sander vitreus)
Source: Animals (Basel). 2024 Aug 28;14(17):2493. doi: 10.3390/ani14172493 (PMC11394479; doi:10.3390/ani14172493)
Supplement: Supplementary file 1 [file animals-14-02493-s001.zip › animals-3115968-supplementary.pdf]

Supplementary

The Effect of the Species Source of Muscle and/or Digestive Enzymes on the Utilization of Fish Protein Hydrolysates as a Dietary Protein Source in First Feed for Larval Walleye (*Sander vitreus*)

Giovanni S. Molinari [1], Michal Wojno [1], Genciana Terova [2], Macdonald Wick [3], Hayden Riley [3], Jeffery T. Caminiti [3] and Karolina Kwasek [1,4,*]

[1] Center for Fisheries, Aquaculture, and Aquatic Sciences, Southern Illinois University, Carbondale, IL 62901, USA; giovanni.molinari@siu.edu (G.S.M.); michal.wojno@siu.edu (M.W.)
[2] Department of Biotechnology and Life Sciences, University of Insubria, 3-21100 Varese, Italy; genciana.terova@uninsubria.it
[3] Department of Animal Science, The Ohio State University, Columbus, OH 44691, USA; wick.13@osu.edu (M.W.); riley.673@osu.edu (H.R.); caminiti.8@buckeyemail.osu.edu (J.T.C.)
[4] Department of Biological Sciences, University of New Hampshire, Durham, NH 03824, USA
* Correspondence: karolina.kwasek@unh.edu; Tel.: +1-(603)-862-2112

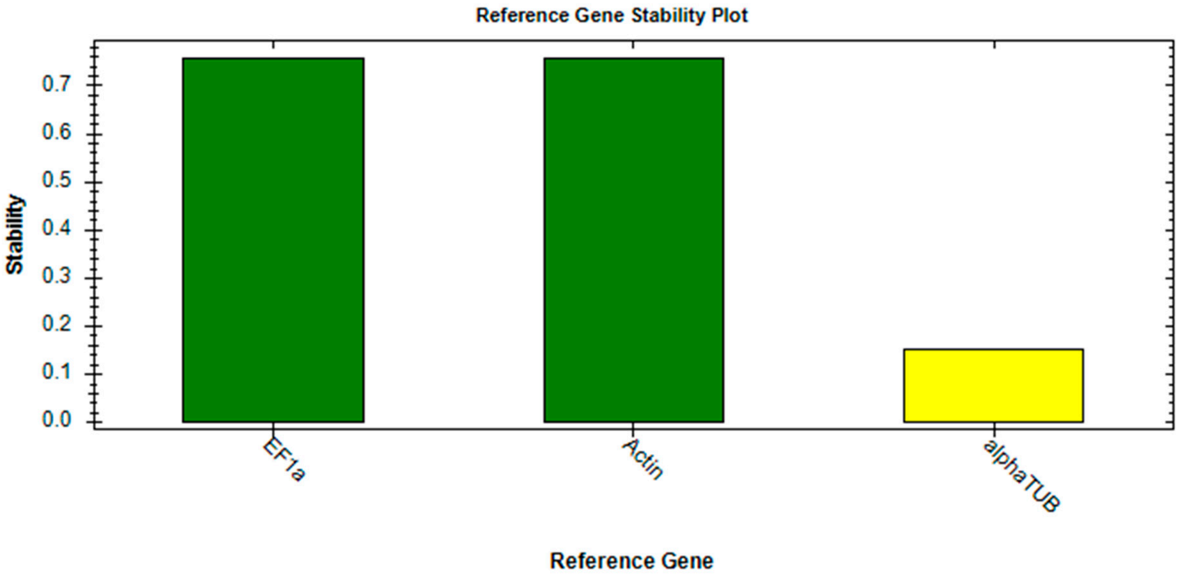

Figure S1. Results of stability analysis of the housekeeping genes *eEF1a1*, *α-tubulin*, and *β-actin.*

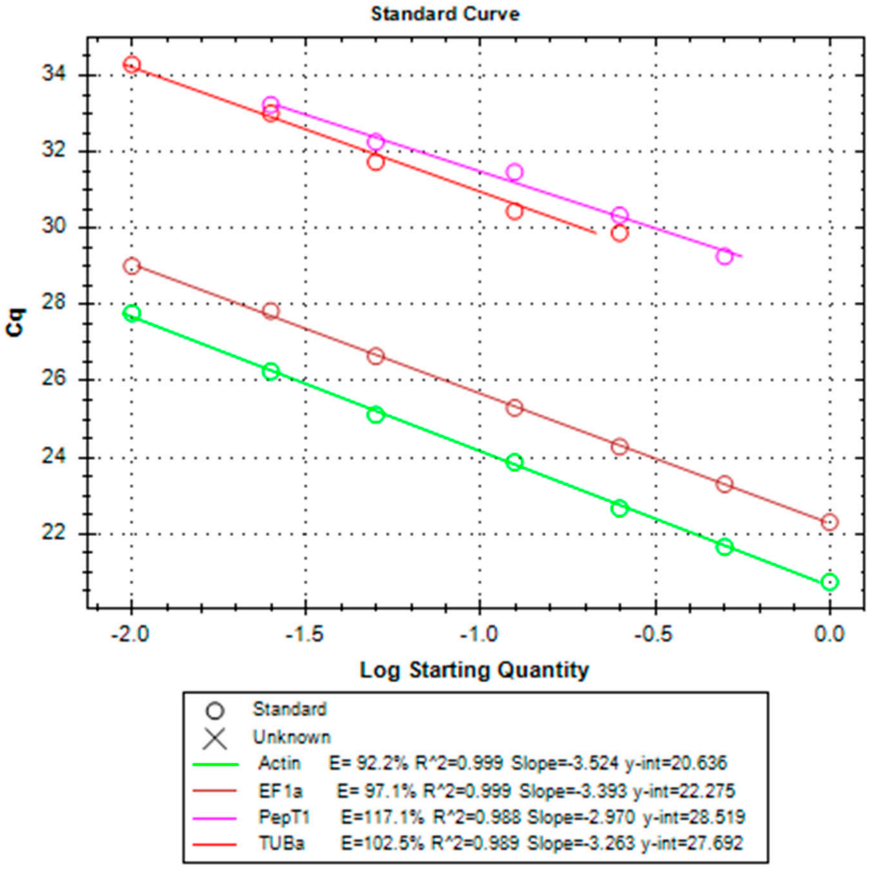

Figure S2. Results of amplification efficiency of each pair of primers for the target and housekeeping genes *eEF1a1*, *α-tubulin*, *β-actin*, and PepT1.
